# Interactions between oxygen homeostasis, food availability, and hydrogen sulfide signaling

**DOI:** 10.3389/fgene.2012.00257

**Published:** 2012-11-27

**Authors:** Nicole N. Iranon, Dana L. Miller

**Affiliations:** ^1^Department of Biochemistry, University of Washington School of MedicineSeattle, WA, USA; ^2^Molecular and Cellular Biology Graduate Program, University of Washington School of MedicineSeattle, WA, USA

**Keywords:** hypoxia, anoxia, oxygen, hydrogen sulfide, suspended animation, diapause, dietary restriction, homeostasis

## Abstract

The ability to sense and respond to stressful conditions is essential to maintain organismal homeostasis. It has long been recognized that stress response factors that improve survival in changing conditions can also influence longevity. In this review, we discuss different strategies used by animals in response to decreased O_2_ (hypoxia) to maintain O_2_ homeostasis, and consider interactions between hypoxia responses, nutritional status, and H_2_S signaling. O_2_ is an essential environmental nutrient for almost all metazoans as it plays a fundamental role in development and cellular metabolism. However, the physiological response(s) to hypoxia depend greatly on the amount of O_2_ available. Animals must sense declining O_2_ availability to coordinate fundamental metabolic and signaling pathways. It is not surprising that factors involved in the response to hypoxia are also involved in responding to other key environmental signals, particularly food availability. Recent studies in mammals have also shown that the small gaseous signaling molecule hydrogen sulfide (H_2_S) protects against cellular damage and death in hypoxia. These results suggest that H_2_S signaling also integrates with hypoxia response(s). Many of the signaling pathways that mediate the effects of hypoxia, food deprivation, and H_2_S signaling have also been implicated in the control of lifespan. Understanding how these pathways are coordinated therefore has the potential to reveal new cellular and organismal homeostatic mechanisms that contribute to longevity assurance in animals.

All organisms must maintain homeostasis to survive. Walter Cannon defined the modern concept of homeostasis as “the coordinated physiological reactions which maintain most of the steady states in the body. . ..” (Cannon, 1929). At the cellular level, maintaining homeostasis requires the coordination of metabolic reactions and cellular processes with environmental conditions. Homeostatic mechanisms are also centrally important for regulating longevity assurance. One consequence of the physiological decline associated with aging is degradation of the ability to maintain homeostasis, which narrows the range of conditions that can be tolerated. At least partly as a result of this defect in homeostasis, the likelihood of death from injury, infection, and disease increases. Oxygen (O_2_) is an essential environmental resource for all metazoans, with only one known exception (Danovaro et al., 2010). The ability to sense and respond to changes in O_2_ likely arose early in evolution (O’Farrell, 2001). Nevertheless, even short exposure to decreased O_2_ availability (hypoxia) leads to irreversible cellular damage and death in most metazoans. Interestingly, responses to hypoxia have molecular and physiological similarities to the effects of food deprivation. Moreover, there is accumulating evidence that hydrogen sulfide (H_2_S) improves outcome after ischemia, suggesting that H_2_S signaling can modulate effects of hypoxia in animals. In this article, we review physiological responses to hypoxia and consider similarities and interactions with adaptation to food deprivation and H_2_S signaling (**Figure 1**).

**FIGURE 1:**
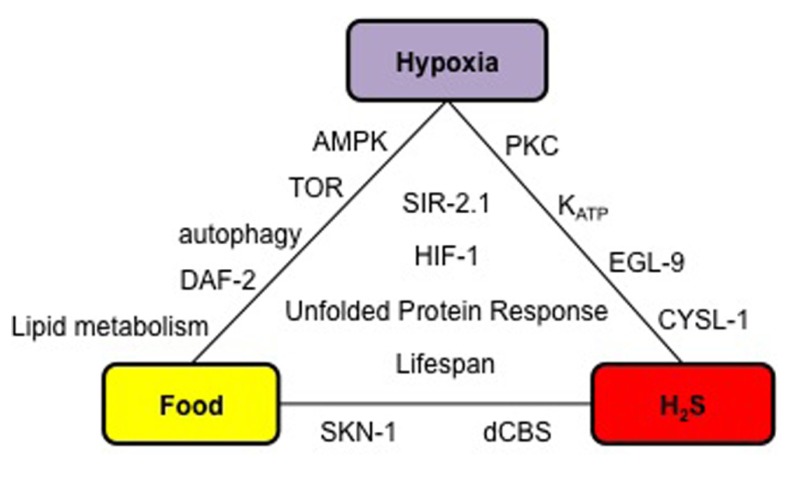
**Physiological and molecular relationships between hypoxia, H_2_S signaling, and food**. Factors listed inside the triangle are common to all three conditions, and those on the edges are shared by two conditions. Details and references are included in the main text.

There is great diversity in sensitivity to hypoxia between different animals and even between cell types in the same animal. For example, hibernating mammals have decreased respiration, with up to 30 min between breaths, and can survive in hypoxic conditions that are damaging to related euthermic non-hibernators (Drew et al., 2004). In global cerebral ischemia, CA1 pyramidal neurons in the hippocampus begin to die before other neurons when blood flow is disrupted (Lipton, 1999). This variation suggests there are mechanisms that promote homeostasis in hypoxia, but that they are only employed in specific physiological contexts. It is important also to consider the precise hypoxic conditions experienced by the cells and organism. The physiological consequences of hypoxia depend greatly on the duration and severity of the hypoxic insult. Hypoxia, where O_2_ levels are “less than normal” or low enough to disrupt normal function, includes a wide range of conditions (**Figure 2**). The ambient concentration of O_2_ at sea level (1 atm atmospheric pressure) is 210,000 ppm (21%) O_2_. At high altitude, though the concentration of O_2_ remains the same, the lower atmospheric pressure results in decreased effective ambient O_2_ tension. O_2_ is poorly soluble in aqueous solutions and diffuses slowly. Therefore, steep O_2_ gradients can exist in poorly mixed water environments and waterlogged soil. It can take >3 h for a 100 mm tissue culture dish to equilibrate with ambient O_2_ levels (Chapman et al., 1970). In large animals, O_2_ is delivered to cells by a complex circulatory system. The concentration of O_2_ at the tissue level is lower than ambient, varies between tissue types, and depends both on O_2_ delivery and tissue metabolic activity (Montgomery, 1957; Dyson and Singer, 2011). Fluctuations in ambient O_2_ supply or tissue metabolic demand stimulate compensatory responses to increase blood flow and O_2_ delivery, including vasodilation, increased respiratory rate, and production of red blood cells. This makes it difficult to experimentally control the hypoxic exposure of cells in an intact animal in order to investigate different cellular responses to hypoxia. It is important also to consider that it is experimentally difficult or impossible to separate damage that occurs in hypoxia or ischemia from effects that occur as a result of reoxygenation. In contrast, *C. elegans *does not have a circulatory system, relying instead on diffusion for O_2_ delivery to cells. This allows for precise experimental control of both genotype and cellular environment (Shen and Powell-Coffman, 2003; Fawcett et al., 2012). Because it is an attractive model for hypoxia research we have built a framework of hypoxia responses as a function of O_2_ tension using *C. elegans*, drawing connections with other systems when possible. There have been several excellent reviews recently about signaling pathways that coordinate cellular responses to hypoxia (Gorr et al., 2006; Powell-Coffman, 2010; Hand et al., 2011; Padilla and Ladage, 2012). In this review we compare how strategies to respond to hypoxia vary with O_2_ concentration, and focus on how response mechanisms could integrate with other signaling pathways to influence organism physiology and lifespan.

**FIGURE 2:**
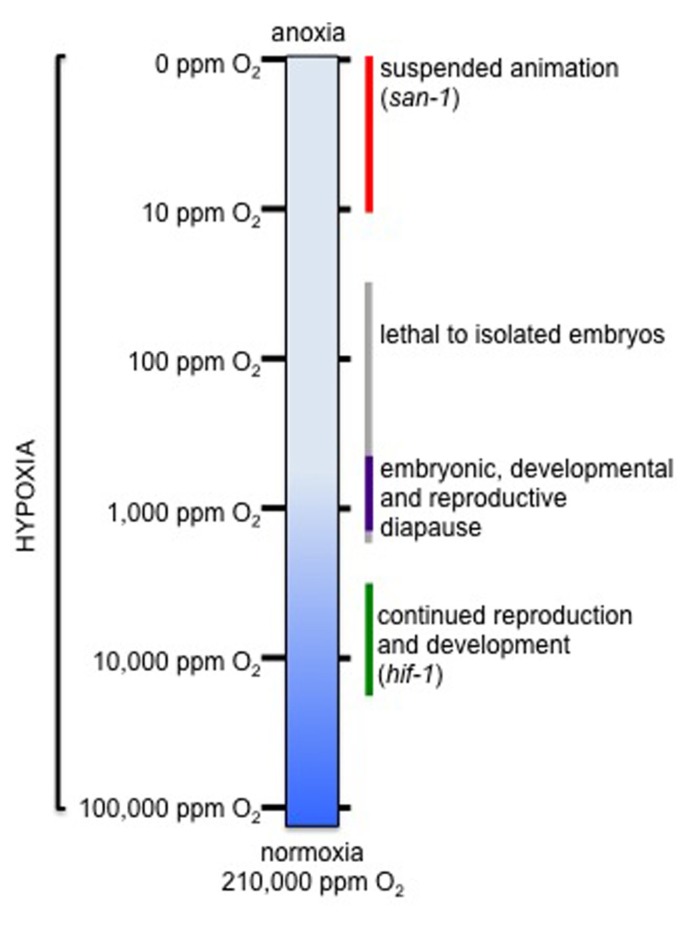
**Hypoxia responses at difference concentrations of O_2_**. The bar represents decreasing O_2_ levels, with normoxia at the bottom and anoxia at the top. For the purposes of this review, normoxia is considered to be room air, which is 210,000 ppm (21%) O_2_. Hypoxia includes all concentrations of O_2_ that are less than this. On the right, the physiological response of *C. elegans* to different O_2_ concentrations is noted, as described in the main text.

## ADAPTATIONS TO ANOXIA

In the laboratory, *C. elegans*, *Drosophila melanogaster*, and *Danio rerio* all survive without O_2_ (anoxia; operationally defined as <10 ppm O_2_) by entering into a state of suspended animation (Foe and Alberts, 1985; DiGregorio et al., 2001; Padilla and Roth, 2001; Padilla et al., 2002). In suspended animation, all microscopically observable activity reversibly arrests, including embryonic cell divisions, post-embryonic development, movement, and reproduction. Upon reoxygenation, developmental processes resume and animals grow to healthy, fertile adults. Suspended animation can be successfully maintained for several days in *C. elegans*, weeks in *Drosophila *embryos, and years in the brine shrimp *Artemia franciscana* (Foe and Alberts, 1985; Clegg, 1997; Padilla et al., 2002). Mechanisms that underlie the ability to survive severe hypometabolic and quiescent states may be widely conserved. Metabolism is dramatically reduced in dogs that survive for several hours after total exsanguination with cold saline flush, for example (Behringer et al., 2003).

One common feature of suspended animation is the reversible arrest of cell divisions. The point at which cell cycle arrest occurs differs between organisms. *C. elegans* embryonic blastomeres arrest in interphase, prophase, and metaphase, but the transition to anaphase will not occur in anoxia (Padilla et al., 2002; Nystul et al., 2003; Hajeri et al., 2005). The spindle assembly checkpoint is activated by anoxia, and stopping the cell cycle is important to prevent lethal chromosome segregation defects. Embryos that have been depleted of *san-1*, a component of the spindle assembly checkpoint, by RNAi die when exposed to anoxia and exhibit chromosome segregation defects (Nystul et al., 2003). In cells that arrest in interphase or prophase, the chromatin condenses and chromosomes align near the nuclear envelope, whereas metaphase blastomeres display reduced spindle and astral microtubule density. The prophase arrest is characterized by inactivation of *cdk-1*, and requires the *npp-16 *nucleoporin (Hajeri et al., 2005). These results indicate that there are at least two distinct cell cycle checkpoints activated to arrest embryonic cell divisions in anoxia-induced suspended animation in *C. elegans*. The spindle assembly checkpoint is not required for suspended animation in adults, possibly because somatic cells are all post-mitotic. However, germline stem cell divisions arrest in adults in suspended animation without any apparent decrease in full reproductive potential (Padilla et al., 2002; our unpublished observation). Thus, there may be other mechanisms that contribute to anoxia-induced suspension of cell division post-embryonically. The mechanisms by which anoxia signaling integrates with the spindle checkpoint are not well understood, though the effect is conserved. *Drosophila *embryos exposed to anoxia also arrest during interphase, prophase, and metaphase, and the arrest is characterized by chromatin localization near the nuclear membrane (Foe and Alberts, 1985;Douglas et al., 2001). Similarly, *Danio rerio *embryos suspend cell division in anoxia, though arrest is exclusively during interphase (Padilla and Roth, 2001).

In anoxia metabolic networks must be substantially rearranged, with important phenotypic consequences. O_2_ is essential for both mitochondrial respiration and fatty acid oxidation. A major consequence of O_2_ deprivation is that cellular energy metabolism is disrupted. The survival of both embryos and adult *C. elegans *in anoxia is correlated with available glycogen stores, which serve as a source for glycolytic energy production (Frazier and Roth, 2009; LaRue and Padilla, 2011). Glycogen decreases progressively as embryos are exposed to anoxia (Frazier and Roth, 2009). Mutations in genes that have little in common, other than decreased glycogen content, all show an anoxia-sensitive phenotype during embryogenesis (Frazier and Roth, 2009). Similarly, hyperosmotic shock, an environmental perturbation that increases glycerol production at the expense of glycogen, reduces the viability of embryos in anoxia (Frazier and Roth, 2009). In contrast, in adults hypomorphic loss-of-function mutations in the insulin/IGF receptor homolog *daf-2 *increase glycogen content and survival in anoxia (Scott et al., 2002; Mendenhall et al., 2006; Frazier and Roth, 2009; LaRue and Padilla, 2011). Diet-induced increases in glycogen are also associated with increased survival in anoxia in *Drosophila *(Vigne et al., 2009). Depletion of the glycolytic enzyme glyceraldehyde 3-phosphate dehydrogenase (*gpd-2/3*) by RNAi decreases survival of adult *daf-2 *mutant animals in anoxia (Mendenhall et al., 2006). The significance of this result is not clear, insofar as *gpd-2/3(RNAi) *does not reduce survival of wild-type animals in anoxia (Mendenhall et al., 2006). One possibility is that the difference between wild-type and *daf-2 *mutant animals reflects a difference in metabolic state. Both gene expression, oxygen consumption measurements, and physiological studies suggest that the *daf-2 *mutant animals have a metabolic architecture that is very different from wild-type (Van Voorhies and Ward, 1999; Lee et al., 2003; Murphy et al., 2003; Houthoofd et al., 2005). Moreover, RNAi directed against other glycolytic enzymes does not alter survival in anoxia (Mendenhall et al., 2006). This may suggest that simply decreasing glycolysis does not explain the effect on anoxia survival. However, it is difficult to assess whether the RNAi treatment sufficiently decreased the activity of the glycolytic enzymes in these experiments, and no direct measurements of effects on glycogen were reported.

In anoxia, fatty acid oxidation is not possible. Instead, increased fatty acid synthesis may be important for anabolic activity and to regenerate reducing equivalents for continued glycolytic activity. Fatty acid synthesis is a hallmark of hypoxic tumor cells (Romero-Garcia et al., 2011), and in *C. elegans *the SREBP homolog *sbp-1 *is required for fatty acid accumulation after anoxia (Taghibiglou et al., 2009). This result suggests that changes in lipid metabolism are essential parts of the response to hypoxia. However, it is also possible that lipid signaling plays an important role during O_2_ deprivation. Consistent with this view, mutations that are predicted to disrupt ceramide synthesis modulate survival in anoxia. Survival was decreased by loss-of-function of *hyl-2*, whereas similar mutations in the related *hyl-1 *increase survival in anoxia (Menuz et al., 2009). In mammalian models, altered ceramide signaling has been associated with hypoxia-induced changes in tumors and may contribute to cell death in neurological disorders including cerebral ischemia (Jana et al., 2009; Yin et al., 2010). *hyl-1* and *hyl-2 *are functional homologs, of LAG1 (longevity assurance gene 1), which was reported to increase replicative lifespan in *Saccharomyces cerevisiae *(D’Mello et al., 1994). However, RNAi knockdown of neither *hyl-1* nor *hyl-2* increase lifespan in *C. elegans *(Menuz et al., 2009). Lipid metabolism and signaling are increasingly recognized as playing an important role in the regulation of aging and lifespan (Lapierre and Hansen, 2012). Considering the important role that aberrant lipid signaling plays in the progression of cancer cells, elucidating the role that these processes play in adaptations to hypoxia is likely to be a productive direction for future research.

There is surprising overlap between genes and pathways that increase survival in anoxia and those that modulate lifespan, though the mechanistic basis of this correlation is not understood. In a screen for genes that increased survival in anoxia when depleted by RNAi, 11 of 198 hits (5.6%) had previously been identified to increase lifespan in *C. elegans *(Mabon et al., 2009). In contrast, the frequency of finding genes that increase lifespan from RNAi screens that use longevity as the primary phenotype ranged from 0.1 to 0.5% (Hamilton et al., 2005; Hansen et al., 2005). Thus, the genes identified by enhanced anoxia survival are enriched for longevity genes. In addition to a variety of metabolic genes identified in this screen, anoxia survival also requires autophagy, which may serve as an important source for catabolic energy production. Disruption of genes important for autophagy by RNAi or mutation reduce survival in anoxia (Samokhvalov et al., 2008). In mammalian systems, autophagy is regulated by hypoxia, particularly in cancer cells (Rouschop and Wouters, 2009; Eskelinen, 2011). Moreover, autophagy is important for increased lifespan by both *daf-2(lf) *loss-of-function mutations and dietary restriction (DR) in *C. elegans *(Meléndez et al., 2003; Hansen et al., 2008). Overexpression of autophagy gene LC3/Atg8 in the nervous system increases lifespan in *Drosophila *(Simonsen et al., 2008). The insulin/IGF1 signaling (IIS) pathway is another conserved pathway that is involved both in longevity assurance and the response to hypoxia. In. *C. elegans*, the IIS receptor homolog *daf-2 *increases lifespan as well as survival in anoxia (Kenyon et al., 1993; Scott et al., 2002; Mendenhall et al., 2006). Increased stress resistance is a well-known feature of *daf-2(lf)* mutant animals, suggesting that increased survival in anoxia is a consequence of a correlation between increased stress resistance and lifespan (Lithgow et al., 1995; Honda and Honda, 1999; Mendenhall et al., 2006; Scott et al., 2002). However, five of six *daf-2 *regulated gene products depleted by RNAi increased resistance to anoxia but had no effect on lifespan (Mabon et al., 2009). Moreover, mutations that increase resistance to osmotic stress, including loss-of-function alleles of *dpy-10 *and *osm-7*, decrease survival in anoxia (Wheeler and Thomas, 2006; Frazier and Roth, 2009). Thus, a general increase in stress resistance does not explain the relationship between lifespan and anoxia resistance.

Protein metabolism is another central aspect of cellular physiology affected by hypoxia. Protein synthesis and the chaperones that help to maintain cellular proteins in the correctly folded state are energetically expensive. The coordination of protein synthesis, quality control, and degradation, referred to as proteostasis, is essential to maintain cellular function (Hartl et al., 2011; Taylor and Dillin, 2011). Reduced protein translation is associated with increased lifespan in *C. elegans *(Hansen et al., 2007; Pan et al., 2007). Many genes that increase survival in anoxia when depleted by RNAi are involved in protein translation. Protein translation is inhibited in low O_2_ (Hochachka et al., 1996; Teodoro and O’Farrell, 2003; Storey and Storey, 2004; Wouters et al., 2005; Liu et al., 2006), making it somewhat surprising that genetic manipulations that decrease translation would increase anoxia survival. It may be that indirect consequences of, or adaptations to, decreased translation confer the protective effect in anoxia. For instance, decreased energy utilization for protein translation could increase energy stores available in anoxia. Another possibility is that reduced translation rates improve proteostasis networks and improve the capacity to deal with unfolded protein stress in anoxia. In the endoplasmic reticulum, the ERO1 enzyme uses O_2_ to catalyze oxidative protein folding (Tu and Weissman, 2002), which would be inhibited in anoxia. In *C. elegans*, the ER unfolded protein response (UPR) is activated in anoxia, and UPR genes *xbp-1* and *ire-1* are required for survival (Mao and Crowder, 2010). This suggests that anoxia increases the burden of misfolded proteins in the secretory path. Decreasing translation by knock-down of aminoacyl tRNA synthase genes reduces expression of UPR mediators, and increases survival in anoxia (Anderson et al., 2009). UPR activity is increased by decreased O_2_ in pancreatic β-cells and liver (but not cardiomyocytes), suggesting that it plays a conserved role in the cellular response to hypoxia (Tagliavacca et al., 2012; Zheng et al., 2012). Understanding general mechanisms that integrate stress homeostasis pathways with the proteostasis network could reveal new strategies to manipulate proteostasis. This would have broad significance, particularly as defects in proteostasis have been associated with the aging process (Haigis and Yankner, 2010; Gidalevitz et al., 2011).

## RESPONSES TO HYPOXIA WHEN SOME O_2_ IS AVAILABLE

A common strategy to survive hypoxia is to avoid conditions with insufficient O_2_. Indeed, animals have evolved sophisticated behavioral strategies to avoid hypoxic conditions. In a gradient of O_2_ blue crabs, New Zealand snapper, and *C. elegans* will all avoid low O_2_ and show preference for an optimal O_2_ environment (Dusenbery, 1980; Bell et al., 2009; Gray et al., 2004; Cook and Herbert, 2012). Interestingly, other environmental conditions can modulate what is perceived as the optimal O_2_ concentration. Hypoxia avoidance in *C. elegans *decreases as animals are starved (Dusenbery, 1980). Both alligators and cold-submerged frogs prefer lower ambient temperature in hypoxia (Branco et al., 1993; Tattersall and Boutilier, 1997). This may reflect a physiological interaction between temperature and O_2_. Consistent with this idea, *C. elegans *survive much longer in anoxia at low temperature than at higher temperature (Padilla et al., 2002; Scott et al., 2002; Mendenhall et al., 2006). It is not clear if the mechanisms that regulate survival are identical in these conditions, though the insulin/IGF receptor ortholog *daf-2* can increase survival at both temperatures (Scott et al., 2002; Mendenhall et al., 2006). The interaction between temperature and hypoxia may also have clinical relevance, as therapeutic hypothermia can reduce neurodevelopmental disability in infants surviving hypoxic ischemic encephalopathy from perinatal asphyxiation, and is used in adults clinically to improve outcome after pelvic surgery, cardiac arrest, and brain ischemia (Selway, 2010; Finley, 2011; Sunde and Søreide, 2011; Yenari and Han, 2012).

In moderate hypoxia (5,000–20,000 ppm O_2_) *C. elegans *embryos complete development and grow to gravid adults, albeit more slowly than in room air (Jiang et al., 2001; Nystul and Roth, 2004; Miller and Roth, 2009). This indicates that the response to these hypoxic conditions is physiologically distinct from anoxia, in which animals enter suspended animation. Consistent with this, embryos do not require *san-1*, the spindle assembly checkpoint protein essential for suspended animation (Nystul and Roth, 2004), to survive exposure to hypoxia. Instead, HIF-1, the single worm homolog of the hypoxia-inducible factor (HIF) is required for embryo survival in 5,000–20,000 ppm O_2_ (Jiang et al., 2001; Nystul and Roth, 2004). HIF is a highly conserved bHLH-PAS domain transcription factor that helps maintain O_2_ homeostasis by coordinating the transcriptional response to hypoxia in metazoans. There are many excellent reviews of HIF function and its role in development and disease (e.g., Semenza, 2009, 2010, 2011, 2012; Majmundar et al., 2010; Powell-Coffman, 2010). HIF was first identified biochemically as the factor that bound the erythropoietin promoter in hypoxia (Wang and Semenza, 1993). HIF is directly regulated by O_2_ levels. HIF is hydroxylated at the conserved proline in the LxxLAP motif by a 2-oxoglutarate-dependent prolyl hydroxylase of the EGLN family, named after *egl-9* in *C. elegans *(Epstein et al., 2001). Hydroxylated HIF is then recognized by an E3-ubiquitin ligase, the Von Hippel–Lindau factor VHL-1, and degraded by the proteasome (Kaelin, 2008). In hypoxia the hydroxylation is inefficient and HIF accumulates, dimerizes with the aryl hydrocarbon nuclear translocator (ARNT; *aha-1*), and induces expression of target genes that facilitate adaptation to hypoxia. In mammals, HIF is essential for early developmental events, and both HIF1α and HIF2α mutant mice die early in embryogenesis (Iyer et al., 1998; Compernolle et al., 2002). HIF homologs are also important for tracheal branching in *Drosophila *and neuronal patterning in *C. elegans*, highlighting the conserved role for HIF in development (Keith and Simon, 2007; Centanin et al., 2008; Pocock and Hobert, 2008). Constitutive stabilization of HIF has been implicated in tumor progression and mutations in VHL, a negative regulator of HIF, are associated with Von Hippel–Lindau syndrome, which is characterized by renal clear cell carcinoma (Kim and Kaelin, 2004; Shen and Kaelin, 2012). Importantly, HIF-1 is not required for embryos to survive suspended animation in *C. elegans*, demonstrating that these two physiological responses to low O_2_ are genetically distinct. Although HIF has been the focus of most studies into transcriptional responses to hypoxia, there is also evidence that other factors are involved. HIF-independent transcriptional responses to hypoxia have been observed in *C. elegans *and mammals (Dong et al., 2001; Shen et al., 2005; Piret et al., 2006; Ndubuizu et al., 2010). The factors that mediate these effects are not well understood.

Despite the fact there are at least two separate adaptive responses to low O_2_ – suspended animation in anoxia or continued development in moderate hypoxia – there are hypoxic conditions that are lethal during embryogenesis. Isolated embryos die when exposed to O_2_ concentrations between 100 and 1,000 ppm O_2_ (Nystul and Roth, 2004). In these conditions, continued developmental progression is associated with increased lethality. Embryos exposed to 1,000 ppm O_2_ undergo more cell divisions and experience a higher rate of lethality than those exposed to 100 ppm O_2_, for 24 h (Nystul and Roth, 2004). Although the cellular mechanisms that underlie these defects are not well understood, it has been demonstrated that inducing suspended animation in isolated embryos using carbon monoxide rescues embryo survival in hypoxia (Nystul and Roth, 2004). Anoxia-induced suspended animation also protects *C. elegans* embryos against otherwise lethal cold exposure (Chan et al., 2010). These results suggest that arresting cell division and development facilitates coordination between cellular events and prevents irrevocable errors. Although embryos cannot autonomously engage suspended animation in these hypoxic conditions, embryos exposed to 1,000 ppm O_2_ *in utero *arrest development and survive (Miller and Roth, 2009). Embryo survival *in utero *requires *san-1*, suggesting that the embryos are in a state genetically related to anoxia-induced suspended animation (Miller and Roth, 2009). We refer to this as a hypoxia-induced diapause, because it is reminiscent of mammalian embryonic diapause, in which the adults remain active but arrest development of embryos *in utero* (Renfree and Shaw, 2000). This embryonic diapause is coordinated by as-yet uncharacterized maternal factors that alter the uterine environment to impinge on embryonic development. Many facets of suspended animation and the mechanisms by which suspended animation can be non-autonomously controlled in the presence of O_2_ remain a mystery and are likely to be a fruitful area of future research.

Developmental context also influences the response to hypoxia, with greater flexibility after embryogenesis. Newly hatched larvae survive in hypoxic conditions that are lethal to embryos (1,000 ppm O_2_), and survival is associated with a reversible arrest of postembryonic development (Miller and Roth, 2009). This suggests that there are mechanisms that can arrest cell division in 1,000 ppm O_2_, but that embryos cannot enact this response. The arrest of post-embryonic cell divisions is genetically distinct from suspended animation, in that *san-1* is not required to arrest cell division of germline stem cells (Miller and Roth, 2009). One caveat to this interpretation is that it has not been demonstrated that *san-1* is required for successful suspension of germline stem cell divisions in adults exposed to anoxia, and it is possible that suspended animation in adults employs different strategies to arrest cell division. Further delineation of the mechanisms used to arrest cell division in these conditions is required to evaluate this possibility. In addition to this developmental arrest, adults exposed to 1,000 ppm O_2_ enter a reproductive diapause (Miller and Roth, 2009). Gravid adults cease laying eggs, arrest the development and fertilization of oocytes, and halt embryonic development *in utero*. The arrest of progeny production ensures that embryos are not produced into conditions where they cannot survive. Moreover, energy shunted away from reproductive activity can be used instead for locomotion to search for a new environment. Therefore, by delaying progeny production animals can find a time and place more suited to successful reproduction. In this way, hypoxia-induced reproductive diapause is similar to diapause in insects and mammals that ensures progeny production is synchronized with seasonal and nutritional conditions that maximize fitness (Renfree and Shaw, 2000; Tatar et al., 2001; Allen, 2007; Guidetti et al., 2008; Tachibana and Watanabe, 2008).

HIF-1 is not required for hypoxia-induced diapause, as animals with a null allele of *hif-1* arrest post-embryonic development and reproduction in 1,000 ppm O_2_ as efficiently wild-type animals (Miller and Roth, 2009). Unlike the situation in embryos, *hif-1(-) *mutant larvae and adults exposed to 5,000 ppm O_2_ survive 24 h with >90% viability to adult upon reoxygenation (Nystul and Roth, 2004; Miller and Roth, 2009). Nevertheless, HIF-1 is necessary for the normal response to 5,000 ppm O_2_. Whereas wild-type animals continue development in these conditions, *hif-1(-)* mutant animals precociously enter into hypoxia-induced developmental and reproductive diapause (Miller and Roth, 2009). This observation supports the idea that responses to hypoxia are specific to the concentration of O_2_ that is available, and that HIF-1 does not play a major role in the response to 1,000 ppm O_2_. In fact, even constitutive activation of HIF-1, by loss-of-function mutations in negative regulator *vhl-1 *or *egl-9*, does not prevent diapause in 1,000 ppm O_2_. This result further suggests that HIF-1 promotes continued developmental activity in both larvae and embryos, though it may have different targets in each developmental context. Although *hif-1 *is expressed in most, if not all, cells (Jiang et al., 2001), expression only in neurons is sufficient to regulate hypoxia-induced diapause in 5,000 ppm O_2_ (Miller and Roth, 2009). This suggests that there are neuroendocrine signaling pathways that coordinate development with the response to hypoxia. In contrast, early stage *hif-1 *mutant embryos die in 5,000 ppm O_2_, suggesting that HIF-1 acts autonomously during embryogenesis, when the nervous system is not fully developed, to protect against hypoxia (Jiang et al., 2001; Nystul and Roth, 2004). The neuronal circuits and neuroendocrine factors that coordinate the systemic response to hypoxia have not been delineated, though it has been shown that hypoxia-induced diapause does not require the same neurons that mediate hyperoxia avoidance behavior (Gray et al., 2004; Miller and Roth, 2009).

The AMP-activated protein kinase (AMPK)is also involved in regulating hypoxia-induced diapause in 5,000 ppm O_2_. AMPK is a conserved serine/threonine kinase that is important for cellular energy homeostasis. In response to disruptions of energy homeostasis, AMPK is activated and phosphorylates targets that increase energy production and decrease energy expenditures (Carling et al., 2011; Hardie, 2011; Mantovani and Roy, 2011; Mihaylova and Shaw, 2011). AMPK is a heterotrimeric protein that consists of a catalytic α subunit and the regulatory β and γ subunits (Hardie et al., 2003). The *C. elegans *genome encodes genes for two AMPK α subunits, *aak-1 *and *aak-2*, two β subunits, *aakb-1 *and *aakb-2*, and five γ subunits, *aakg-1–5* (Beale, 2008). In 1,000 ppm O_2_ *aak-2(lf) *mutant animals are fully capable of entering into and surviving diapause. However, *aak-2(lf)* mutant animals precociously enter diapause in 5,000 ppm O_2_ (Miller and Roth, 2009). Thus, like HIF-1, AAK-2 acts to oppose diapause in hypoxia and support continued developmental activity. AAK-2 is not required for embryonic or larval survival in either 1,000 or 5,000 ppm O_2_ (Miller and Roth, 2009), though it is required for long-term survival in anoxia (LaRue and Padilla, 2011). The source of this discrepancy could be either the duration or severity of O_2_ deprivation. Another possibility is that AMPK has different function in different hypoxic conditions. This could result if different AMPK complexes are active in each O_2_ concentration. In addition to *aak-2, aakb-1/2* and *aakg-2 *contribute to long-term survival in anoxia (LaRue and Padilla, 2011). It is not known which subunits other than *aak-2* are involved in coordinating hypoxia-induced diapause. Another possibility is that different AMPK substrates mediate these different physiological effects, depending on context.

The mechanisms by which AMPK integrate with cellular and developmental functions to regulate hypoxia-induced diapause have not been defined. Full activation of AMPK requires phosphorylation of the α subunit by an activating kinase. Genetic studies suggest there are at least three kinases upstream of AMPK, including LKB1 (*par-4*), Ca^2^^+^/calmodulin-dependent protein kinase kinase (*ckk-1*), and the MAP kinase kinase kinase TAK1 (*tap-1*; Carling et al., 2008; www.wormbase.org WS231). AMPK is also stimulated by AMP (which increases when ATP levels fall), but in mammalian systems hypoxia activates AMPK independent of ATP levels (Laderoute et al., 2006; Liu et al., 2006; Papandreou et al., 2008). The importance of these upstream kinases in different hypoxia contexts has not been investigated. In mammalian cells, activation of AMPK by hypoxia is abrogated by depletion of CAMKKβ but not LKB1 (Mungai et al., 2011). The role of TAK1 in regulating AMPK homologs in animals is still a matter of investigation. Recent proteomic studies have revealed that AMPK directly phosphorylates many components of the cell cycle machinery (Banko et al., 2011). These studies suggest a preliminary model in which HIF-1 acts upstream or in parallel to AMPK, which regulates cell division in hypoxia. Working out the mechanistic details that govern this effect is likely to provide unique insight into how AMPK coordinates cellular activities in response to metabolic stress.

## RELATIONSHIP BETWEEN HYPOXIA AND FOOD DEPRIVATION

Hypoxia and food deprivation are similar stresses in that they both affect central aspects of cellular metabolism. The absence of either food or O_2_ disrupts energy-generating pathways, and there are similarities in physiological responses and molecular genetic pathways that are activated in these two situations. The integration of these pathways is highlighted by the interactions between hypoxia and nutrient availability. Rats that are subject to alternate-day feeding have reduced neuronal damage and improved behavioral outcomes after focal cerebral ischemia (Yu and Mattson, 1999). Similarly, mice that are fasted for only 3 days are resistant to surgically induced renal and hepatic ischemia/reperfusion (I/R) injury (Mitchell et al., 2010; Verweij et al., 2011). In contrast, both severe and moderate food restriction decrease survival after gut I/R from occlusion of the superior mesenteric artery (Ueno et al., 2005). Given the therapeutic potential, there is much interest in understanding the mechanistic basis of the interaction between fasting and hypoxia and I/R.

In both* C. elegans *and *Drosophila* exposure to hypoxia increases lifespan, though the relationship is not linear and different hypoxic conditions increase lifespan in these species (Honda et al., 1993; Mehta et al., 2009; Rascón and Harrison, 2010). Decreased food intake, DR, also increases lifespan in these and other species (Koubova and Guarente, 2003; Bishop and Guarente, 2007; Fontana et al., 2010), though there are some genetic backgrounds and species in which DR does not increase lifespan (Mockett et al., 2006; Swindell, 2012). Many genetic pathways that are involved in mediating the effects of DR on lifespan also have roles in the response to hypoxia, and vice versa. This suggests that responses to DR and hypoxia may physiologically interact as well. Indeed, dietary conditions that maximize lifespan are different for *Drosophila* in hypoxia and normoxia. Flies chronically adapted to 50,000 ppm O_2_ live longer at lower yeast (protein) levels than normoxic cohorts (Vigne and Frelin, 2007). In *C. elegans* the IIS pathway downstream of *daf-2*, *aak-2*, and the target of rapamycin (TOR) kinase *let-363* have all been shown to be important for increased lifespan in DR (Greer and Brunet, 2009). As noted above, both *daf-2* and AMPK are important in mediating responses to decreased O_2_. Although a role of TOR/*let-363 *in *C. elegans *hypoxia response has not been demonstrated, TOR is negatively regulated by AMPK, and TOR mediates the translational arrest observed in mammalian cells exposed to hypoxia (Liu et al., 2006; Lee et al., 2008). This suggests the possibility that these factors mediate increased lifespan in response to both decreased food and hypoxia.

HIF-1 has recently been shown to modulate lifespan in *C. elegans*. Curiously, both *hif-1(-) *and *vhl-1(-)* mutant animals, which have constitutively stabilized HIF-1, exhibit increased lifespans (Chen et al., 2009; Mehta et al., 2009; Zhang et al., 2009). It may be that different environmental contexts underlie this effect. The *hif-1* mutant was subsequently shown to be long-lived at low temperature but not at high temperature (Leiser et al., 2011). HIF-1 is required for *C. elegans *to adapt to changes in temperature (Treinin et al., 2003), and HIF is stabilized in both crucian carp and mice exposed to high temperature (Katschinski et al., 2002; Rissanen et al., 2006). Thus, HIF may have an important role in responding to thermal stress as well as hypoxia. Notably, the *hif-1(-)* mutant animal does not have increased lifespan in DR. While *C. elegans *that overexpress HIF-1 due to a mutation in *egl-9* show modest increases in lifespan under DR, the effect is blunted compared to wild-type animals (Chen et al., 2009). These results suggest that *hif-1 *may be generally involved the response to decreased food and well as decreased O_2_. Longevity mediated by both DR and mutation of *hif-1* require the ER stress signaling genes *ire-1* and *xbp-1*, which function to activate the UPR (Chen et al., 2009), suggesting an interaction between ER stress and nutrient sensing. In mice HIF is stabilized by glucose in POMC neurons in the hippocampus, and plays a role to regulate feeding and organismal energy balance (Zhang et al., 2011). Together, these observations suggest that HIF may coordinate a conserved integration of nutrient sensing with hypoxia. It will be important to further understand the mechanisms by which these response pathways to determine if this is a direct effect.

Developmental arrest is a common response to both food deprivation and hypoxia in *C. elegans*. Larvae that hatch in conditions without food do not initiate post-embryonic development and can persist for weeks in this state, referred to as the L1 diapause. Similarly, if food deprivation occurs in the last larval stage, L4, animals can enter into an adult reproductive diapause that is characterized by the arrest of oocyte production and fertilization (Angelo and Van Gilst, 2009). The arrest observed in hypoxia-induced diapause is superficially similar to L1 diapause, as both somatic and germline development arrest in animals on food in 1,000 ppm O_2_. However, there are differences in the genetic factors required in each situation. Neither *daf-16*, the FOXO transcription factor downstream of the IIS pathway, nor the PTEN homolog *daf-18* is required for larvae to reversibly arrest development and survive for 24 h in 1,000 ppm O_2_ (Miller and Roth, 2009). In contrast, loss-of-function mutations in *daf-16 *or *daf-18 *abrogate the ability to maintain developmental arrest and survive food deprivation (Baugh and Sternberg, 2006; Fukuyama et al., 2006). This discrepancy suggests that L1 arrest involves different mechanisms in each condition, though it is possible that the difference stems from longer duration of arrest in the starvation experiments. Another feature that distinguishes hypoxia-induced diapause from starvation is that hypoxia can arrest development at any point, whereas there seem to be specific points in development in which food withdrawal can cause developmental arrest (Angelo and Van Gilst, 2009; Miller and Roth, 2009; Seidel and Kimble, 2011). This difference may stem from the fact that O_2_ must be continuously acquired from the environment whereas fats, proteins, and sugars can be stored for later use. After extended periods of starvation in the adult reproductive diapause the germline retracts until only a small population of stem cells remains (Angelo and Van Gilst, 2009). The nuclear hormone receptor *nhr-49 *is required to appropriately enter into starvation-induced adult reproductive diapause. In contrast, the germline remains intact in hypoxia, and suspension of reproduction does not require *nhr-49 *(Miller and Roth, 2009 and our unpublished observation). As in hypoxia, when gravid adults are removed from food they arrest egg-laying. In starved adults embryo development *in utero *continues until the progeny hatch and devour the adult from within, a process known as “bagging” or facultative vivipary (Chen and Caswell-Chen, 2004; Schafer, 2005). The arrest of embryo production in development in hypoxia prevents bagging, however. It has been reported that embryos also arrest in the uterus of adults in starvation-induced adult reproductive diapause (Angelo and Van Gilst, 2009), though this result has been recently questioned (Seidel and Kimble, 2011).

If food is scarce in development, *C. elegans *will enter an alternative larval stage called dauer, where development arrests until conditions improve. High temperature and crowding also influence the dauer decision. Developmental arrest in dauer is regulated by neuroendocrine signals as well as the IIS and TGFβ signaling pathways (Hu, 2007; Fielenbach and Antebi, 2008). The IIS pathway does not have an apparent role in hypoxia-induced diapause (Miller and Roth, 2009). However, some genes regulated by hypoxia are also regulated by entry into dauer, and at high temperature*hif-1(-) *mutant animals arrest as partial dauers (Shen et al., 2005). This suggests that there is cross-talk between the IIS pathway and *hif-1*. Similarly, AMPK is required for normal response to hypoxia and in dauer. In dauer, germ cell divisions do not arrest appropriately in *aak-2 *mutant animals (Narbonne and Roy, 2006). Thus, in contrast to hypoxia, where AAK-2 promotes cell division and development, in dauer it is required to arrest of germline cell divisions. This observation further suggests that AMPK has different roles in regulating developmental progression in specific physiological contexts.

## INTERACTIONS BETWEEN H_2_S SIGNALING AND HYPOXIA

Emerging evidence suggests that H_2_S signaling can modulate the physiological effects of hypoxia in mammals. H_2_S is naturally produced in animal cells as a product of amino acid metabolism though the transsulfuration pathway (Dominy and Stipanuk, 2004; Stipanuk, 2004). Endogenously produced H_2_S has many important roles in cellular signaling, neuromodulation, and regulation of vascular tone (Kimura, 2011; Vandiver and Snyder, 2012; Wang, 2012). At low concentrations exogenous H_2_S has dramatic physiological effects that improve survival in changing conditions. Mice exposed to 80 ppm H_2_S, in otherwise normal room air, enter into a suspended-animation-like state in which basal metabolic rate is depressed and core body temperature is maintained only slightly above ambient (Blackstone et al., 2005; Volpato et al., 2008). Mice exposed to low H_2_S survive in otherwise lethal hypoxia (Blackstone and Roth, 2007), and H_2_S improves outcome in a variety of mammalian models of I/R spanning multiple organ systems, including myocardial infarct, hepatic I/R, and lung injury from smoke inhalation (Szabó, 2007; Nicholson and Calvert, 2010; King and Lefer, 2011).

The mechanisms by which H_2_S signaling integrates with hypoxia are not well understood. Pharmacological inhibitors of K_ATP_ channels and protein kinase C (PKC) abrogate the protective effect of NaHS, the ionized form of H_2_S, in a neuronal cell culture model of hypoxic injury (Tay et al., 2010). Similarly, the vasodilatory effects of NaHS depend partially on plasma membrane K_ATP_ subunit SUR2 (Liang et al., 2011). The ability for H_2_S to stimulate rat K_ATP_ channels heterologously expressed in HEK293 cells requires specific cysteine residues (Jiang et al., 2010), suggesting that H_2_S directly sulfhydrates the K_ATP_ channel to modulate its activity. However, endogenously produced H_2_S post-translationally modifies up to 80% of cellular proteins (Mustafa et al., 2009), and elucidating the functionally relevant targets of H_2_S in different contexts is a major challenge. In addition to K_ATP_ channels, H_2_S has been proposed to directly activate mitochondrial energy production in smooth muscle of mice (Fu et al., 2012). Similar activity has been reported for ciliated mussel gills (Doeller et al., 1999) and isolated chicken liver mitochondria (Yong and Searcy, 2001), suggested that the ability to stimulate cellular energy production may be a conserved features of H_2_S (Theissen et al., 2003; Olson, 2012). Cardioprotective effects of H_2_S administration in murine models of myocardial ischemia require the Nrf2 transcriptional factor (Calvert et al., 2009, 2010). *C. elegans *require the Nrf2 homolog *skn-1* to survive H_2_S, and some early transcriptional changes in H_2_S depend on *skn-1* (Miller et al., 2011). SKN-1 is important for the response to various oxidative stresses, though the gene products that are regulated can vary depending on context (An and Blackwell, 2003; Oliveira et al., 2009; Li et al., 2011). SKN-1 is required for increased stress resistance and lifespan resulting from inhibiting either TOR or IIS (Tullet et al., 2008; Robida-Stubbs et al., 2012), and it is also required in the two ASI neurons for increased lifespan by DR (Bishop and Guarente, 2007).

The transcriptional response to H_2_S requires *hif-1* in *C. elegans*, suggesting a potential mechanistic link between the response to hypoxia and H_2_S. HIF-1 is stabilized and accumulates in the nucleus upon exposure to H_2_S in *C. elegans *(Budde and Roth, 2010). Similarly, NaHS induces expression and accumulation of HIF in rat endothelial cells (Liu et al., 2010). Increased expression of *hif-1 *target genes and survival in H_2_S requires CYSL-1, which binds to EGL-9 and is proposed to inhibit its ability to hydroxylate HIF-1 (Budde and Roth, 2010; Ma et al., 2012). CYSL-1 is member of the cystathionine β-synthase/cysteine synthase family of pyridoxal-5′-phosphate (PLP)-dependent enzymes that has *O*-acetylserines ulfhydrylase activity *in vitro *(Ma et al., 2012). All of the transcripts that accumulate after 1 h exposure to H_2_S require *hif-1 *(Miller et al., 2011). However, it is not yet clear how H_2_S effects on HIF contribute to protection in hypoxia. The *hif-1*-mediated response is essential for animals to survive exposure to H_2_S (Budde and Roth, 2010), whereas *hif-1(ia04) *mutant animals can survive 24 h exposure to hypoxia (Miller and Roth, 2009). Moreover, there is curiously little overlap between gene products that require *hif-1 *to accumulate in response to hypoxia and H_2_S (Miller et al., 2011). The source of this variation has not been determined, but could reflect different tissues of activity, other cooperating transcription factors, or context-dependent effects on HIF-1 activity deriving from other signaling events.

H_2_S increases lifespan and thermotolerance in *C. elegans *(Miller and Roth, 2007), and overexpression of dCBS, a H_2_S-producing enzyme in the transsulfuration pathway, modestly increases lifespan in *Drosophila *(Kabil et al., 2011). Pharmacological inhibition of dCBS and RNAi-mediated knockdown of dCBS abrogates increased lifespan by DR (Kabil et al., 2011). These experiments suggest the possibility that H_2_S signaling also integrates with nutrient sensing pathways. In *C. elegans *the effects of H_2_S on lifespan require the conserved sirtuin, *sir-2.1 *(Miller and Roth, 2007). Sirtuin activity is intricately linked with metabolic adaptations to stress, as its activity can be modulated by changes in redox state and metabolic status (Yang et al., 2006; Schwer and Verdin, 2008; Weyrich et al., 2008; Longo, 2009; Yu and Auwerx, 2009; Donmez and Guarente, 2010; Haigis and Yankner, 2010). In mammals, the SIRT1 sirtuin deacetylates and activates HIF (Lim et al., 2010). This suggests the possibility that *sir-2.1 *activates *hif-1*, leading to physiological responses to H_2_S that increase lifespan.

## CONCLUSION

Signaling pathways that mediate responses to decreased O_2_, food deprivation, and H_2_S are integrated with fundamental aspects of cellular physiology and metabolism. As a result, these (and other) stress responses depend on the initial state of the organism. Anything that changes the physiological state – such as aging or previous stress exposure – will necessarily change response(s) to subsequent stresses. In this way, stress responses can be considered to be path dependent: the initial conditions determine the magnitude and trajectory of the response. A greater understanding of the systems biology of stress responses will provide insight into how physiological systems change with age, and may suggest new strategies to delay age-associated disruptions in homeostasis.

Many important questions remain to be answered that will advance our understanding of mechanisms that underlie how context-dependent stress responses are coordinated. For example, we understand little about how conserved factors such as AMPK and HIF have different effects in different conditions. The physiological basis for H_2_S signaling effects physiological functions, including lifespan and stress response are relatively unexplored. Similarly, the mechanisms by which proteostasis networks are integrated with conditional stress responses are not well understood. In order to address questions requires that both genetic and environmental conditions can be precisely controlled experimentally. The power of genetically tractable model organism systems provides great promise in this regard, as do unbiased approaches that have the potential to reveal novel regulators in these responses. Moreover, these studies will reveal neuroendocrine signaling factors that coordinate the organism-wide response to changing conditions.

Insufficient or inappropriate responses to hypoxia contribute to the progression of many human diseases, suggesting that it may be possible to exploit context-dependent physiological responses for clinical benefit. For example, the observation that the fasting response protects normal cells from chemotherapeutic agents more than cancerous cells led to the simple idea of using fasting to improve the efficacy of chemotherapeutics (; Lee et al., 2012). This promising study demonstrates the importance of understanding how diverse stress responses are coordinated with each other and is an excellent example of the promise of this emerging research area.

## Conflict of Interest Statement

The authors declare that the research was conducted in the absence of any commercial or financial relationships that could be construed as a potential conflict of interest.
